# Evaluating the effects of stocking density on the behavior, health, and welfare of turkey hens to 11 weeks of age

**DOI:** 10.1016/j.psj.2022.101956

**Published:** 2022-05-06

**Authors:** S. Jhetam, K. Buchynski, T. Shynkaruk, K. Schwean-Lardner

**Affiliations:** Department of Animal and Poultry Science, University of Saskatchewan, Saskatoon, SK, Canada, S7N 5A8

**Keywords:** footpad lesions, mobility, feather cover, aggression, stress

## Abstract

Nicholas Select hens (n = 3,550 poults in each of 2 experimental trials) were randomly placed in 1 of 4 stocking density (**SD**) treatments of 30, 40, 50, or 60 kg/m^2^ until 11 wk. Birds were housed in open rooms (67.5 m^2^) with 4 replications per treatment. Ventilation was adjusted in each room independently to ensure air quality measures did not differ across replicate rooms. At wk 8 and 11, footpad lesions, mobility, feather cover and cleanliness, behavior (recorded), and litter moisture were evaluated. Incidences of aggressive pecking were recorded daily. Heterophil/lymphocyte (**H/L**) ratios were evaluated at 3, 5, 8, and 11 wk. Data were analyzed using regression analyses in SAS 9.4 (Proc Reg and Proc RSReg; SD as independent variable). Differences were considered significant when *P* ≤ 0.05. Gait scores were not affected by SD. Average footpad scores worsened with increasing SD at wk 8 (linear) but were not affected at wk 11. Total feather cover scores and average feather cleanliness were poorer at high SD (linear) at wk 8 and 11. The incidence of aggressive pecking and culls for aggressive damage decreased linearly as SD increased. At 5 (linear) and 11 (quadratic) wk, H/L ratios increased as SD increased. At 8 wk, H/L ratios were highest in the 40 kg/m^2^ treatment (quadratic). At 8 wk, the percentage of birds at the feeder, resting, and total disturbances linearly increased as SD increased. The percentage of birds standing, walking, and litter pecking decreased linearly with increasing SD, while total aggressive behaviors (sum of fighting and aggressive pecking) decreased (quadratic). At 11 wk, the percentage of birds at the drinker, and decreased with increasing SD while resting, feather pecking, and severe disturbances increased as SD increased. Litter moisture increased linearly with increasing SD (wk 11). Turkey hen health and welfare were negatively impacted by higher SD. At low SD, there was notably more aggression which may also impact welfare.

## INTRODUCTION

Welfare implications of how livestock are raised have become increasingly important to consumers, however, it is often difficult to quantify. Welfare is commonly assessed by breaking down the evaluation into categories of biological functioning, affective states, and ‘natural’ living (Fraser, 2008). Understanding why birds perform specific behaviors in intensive production systems can aid in welfare evaluations and combining physiological parameters with behavior can help determine if a bird's wellbeing is affected. Stocking density (**SD**) is a complicated parameter of poultry management that can have varying effects on the production, health, and welfare of turkeys. The Canadian Codes of Practice recommend a maximum SD range from 40 to 65 kg/m^2^ depending on the final predicted BW of the turkeys raised (National Farm Animal Care Council, 2016). For turkeys raised between 6.2 and 10.8 kg, such as the hens in this study, the recommended SD is 45 kg/m^2^ up to 50 kg/m^2^ if specific environmental and management requirements are met (National Farm Animal Care Council, 2016). However, there are many differences found in SD recommendations between countries which may be a result of the varying effects of SD on turkeys reported in previous literature. Determining the relationship between SD and bird wellbeing is complicated but necessary to make appropriate science-based recommendations.

The majority of the SD research has been done with broiler chickens, however, some turkey specific research has been performed. The health and welfare of turkeys raised at high and low SD has previously been evaluated by examining footpad lesions, gait scores, feather condition, stress, and behavior (Martrenchar et al., 1999; Buchwalder and Huber-Eicher, 2004; Gunther and Bessei, 2006; Beaulac and Schwean-Lardner, 2018). In the few turkey SD studies published, authors often included only one or two health parameters and more often focused on performance measures (Denbow et al., 1984; Leighton et al., 1985; Martrenchar et al., 1999; Hafez et al., 2016).

This study was part of a larger study that included evaluating the effects of SD on turkey hen performance (Jhetam et al., 2022) in addition to health and welfare parameters. Jhetam et al. (2022) observed a linear decrease in BW at 11 wk with increasing SD (30–60 kg/m^2^) and there was a trend observed for overall BW gain (0–11 wk) to decrease with increasing SD. Feed consumption decreased as SD increased from 8 to 11 wk and for the duration of the trial from 0 to 11 wk. However, feed efficiency, mortality, and flock uniformity were not affected by SD. Although high SD negatively affected some performance parameters in this study, low SD resulted in increased aggression related mortality. Therefore, it is important to assess health and welfare parameters in addition to performance when developing SD guidelines.

Footpad dermatitis (**FPD**), mobility, and feather condition are important measures when determining turkey wellbeing in relation to SD. Footpad lesions have been linked to poor gait and could indicate discomfort and pain (Martland, 1984; Weber Wyneken et al., 2015). Bird mobility, evaluated by subjective gait scoring, is a measure of bird welfare because when a bird's mobility is reduced, it can impact growth by limiting access to feed and water and may cause pain from skeletal abnormalities in more severe cases of reduced mobility (Jong et al., 2012; Jankowski et al., 2015). Studies have found an increase in the incidence of FPD with increasing density (Martrenchar et al., 1999; Beaulac and Schwean-Lardner, 2018). Martrenchar et al. (1999) found turkey hens to have poorer mobility (assessed via gait scores) at higher SD (62.7 kg/m^2^) at 12 wk of age and the same trend was found in toms at 16 wk of age, which correlated to the presence of FPD at that age (Beaulac and Schwean-Lardner, 2018). Poor feather cover can be an indicator of bird welfare when poor feather cover is caused by feather pecking which damages or removes feathers or when abrasive damage occurs (Beaulac and Schwean-Lardner, 2018). At higher densities (48 kg/m^2^), feather cover was poorer in 14-wk-old hens (Coleman and Leighton, 1969) and both feather cover and cleanliness were poorer in 16-wk-old toms when housed at high SD (60 kg/m^2^) (Beaulac and Schwean-Lardner, 2018).

The ratio of heterophils to lymphocytes (H/L ratio) has previously been used to assess chronic stress. Measuring H/L ratios has been verified as a quantitative and reliable method of evaluating bird wellbeing, specifically stress related to social and environmental stressors (Gross and Siegel, 1983; McFarlane and Curtis, 1989). Beaulac and Schwean-Lardner (2018) evaluated H/L ratios at 4, 12, and 16 wk of age in toms, and H/L ratios were higher with increasing SD at 4 wk of age. The increased stress response found at 4 wk of age corresponded with numerical increases in aggressive damage up to 4 wk of age. Between 4 and 8 wk of age, a quadratic relationship was observed for aggressive damage with the lowest (30 kg/m^2^) and highest (60 kg/m^2^) SD treatments having the highest incidence of aggressive damage. Aggressive pecking causing damage to the skin is a welfare concern as it is painful and could require to culling of otherwise healthy turkeys (Buchwalder and Huber-Eicher, 2003; Beaulac and Schwean-Lardner, 2018).

Behavior studies in relation to SD have been limited in turkey hens, and few have been conducted with turkey toms. Contradictory behavioral results are common in SD studies due to confounding factors. There are significant variations in group sizes, pen sizes, age of turkeys, bird sex, and environmental conditions utilized in the various published studies, making comparisons difficult. For example, some studies have shown increased aggression with increasing density (Buchwalder and Huber-Eicher, 2004; Beaulac and Schwean-Lardner, 2018). However, older studies found no increase in aggression with increasing SD levels (Leighton et al., 1985; Martrenchar et al., 1999). It was found that bird activity was reduced in higher SD treatments in some studies (Gunther and Bessei, 2006; Beaulac and Schwean-Lardner, 2018), whereas others found no effect of SD on walking or resting activity (Martrenchar et al., 1999).

Few studies have evaluated the specific effect of SD on turkey hen behavior, stress, and other health parameters, and many of those studies were performed over 10 yr ago. Therefore, more research is necessary to determine an appropriate SD that does not negatively affect bird health and welfare. The objectives of this study were to evaluate the effects of SD on turkey hen health and welfare to 11 wk of age while reducing confounding factors including air quality (carbon dioxide and ammonia) and feeder and drinker space. It was hypothesized that increasing SD would negatively impact the health of turkey hens resulting from increased incidence of FPD, poorer mobility, poorer feather coverage and cleanliness, and increased stress. Explanation for these impacts may include increased litter moisture and less available space for mobility behaviors such as walking at higher densities. It was also hypothesized that low SD levels would result in more aggression due to disturbances from other birds and increased activity.

## MATERIALS AND METHODS

This study was carried out in accordance with the recommendations of the Guide to the Care and Use of Experimental Animals and the CCAC guidelines on the Care and Use of Farm Animals in Research, Teaching and Testing, Canadian Council on Animal Care (Canadian Council on Animal Care, 2009). The protocol was approved by the University of Saskatchewan's Animal Care Committee (AUP 19940248). The primary objective of this manuscript focused on the effects of SD on turkey hen behavior, health, and welfare, however, the data regarding the effects of SD on turkey hen performance and environmental conditions have also been reported (Jhetam et al., 2022). A summary of basic performance data is provided in Table 1 for reference.

**Table 1:** Summary of estimated final stocking density effects on turkey hen growth, feed consumption, feed efficiency, and mortality from Jhetam et al. (2022).

Performance Parameter	n	Stocking density (kg/m^2^)				SEM^1^	*P*-value (Linear)	*P*-value (Quadratic)	Regression Equation^2^
		30	40	50	60				
**Body weight (kg)**									
Wk 11	4	8.36	8.35	8.30	8.19	0.033	0.05	0.44	Y = -0.56e^−2^x+8.55
**Body weight gain (kg)**									
Wk 0-11	4	8.31	8.29	8.25	8.13	0.033	0.06	0.44	-
**Feed consumption (kg)**									
Wk 8-11	4	7.50	7.47	7.38	7.21	0.041	<0.01	0.32	Y = -0.94e^−2^x+7.81
Wk 0-11	4	15.19	15.29	15.08	14.90	0.055	0.01	0.04	Y = -0.88e-^3^x^2^+0.067x+14.00
**Feed-to-gain ratio (mortality corrected)**									
Wk 0-11	4	1.90	1.92	1.90	1.91	0.024	0.95	0.87	-
**Mortality and culls (%)**									
Wk 0–11	4	8.05	7.47	8.61	8.49	1.592	0.86	0.926	-

1 Standard error of the mean.

2 Regression considered significant if *P* ≤ 0.05.

### Experimental Design

This experiment was conducted at the University of Saskatchewan Poultry Centre in a floor facility containing individual rooms, each of which can be controlled independently for environmental conditions. This experiment was a randomized complete block design with 2 trials (trial as the block) to allow for increased replication. Each treatment was randomly assigned to 1 of 8 rooms (replicate unit) allowing for 2 room replications per treatment per trial for a total of 4 replications per treatment. The 4 target stocking densities (at 11 wk) were 30, 40, 50, and 60 kg/m^2^ and parameters were evaluated from placement (d 0) to 11 wk of age. The average actual SD achieved at 11 wk was 31.70, 42.38, 52.01, and 61.33 kg/m^2^.

### Birds and Housing

A total of 3,550 Nicholas Select turkey hens were placed in each trial. The poults were obtained from a commercial hatchery, where their beaks and front 3 toes were infrared treated. The birds were randomly selected and assigned to a treatment with an additional 5% of birds placed to account for predicted mortality, allowing for the final target SD to be achieved. The number of birds placed in each treatment was calculated according to the final predicted BW (7 kg) of turkey hens at 11 wk of age (Aviagen, 2015a) (295, 388, 482, and 571 birds per room for the final predicted SD treatments of 30, 40, 50, and 60 kg/m^2^, respectively).

Birds were housed in 1 of 8 large open rooms (6.71 × 10.06 m; 67.50 m^2^) in each trial. Each treatment was replicated twice per trial (n = 4). Birds were brooded on wood shavings 7 to 10 cm thick for the first 10 d, followed by wheat straw (depth of 10–13 cm) for the rearing period. Brooder rings, 7.0 m in diameter, and heat lamps were used for the first 10 d, and wood shavings in the brooder ring area were top-dressed with wheat straw on d 10. Birds were fed ad libitum using aluminum tube feeders with a pan diameter of 36 cm for the first 40 d and 44 cm for the remainder of the trial. Water was provided via Lubing EasyLine pendulum turkey nipple drinkers (Lubing, Cleveland, TN). Feeder and drinker space were equalized on a per bird basis for each SD treatment (35 birds/feeder; 30 birds/nipple), thereby eliminating impacts of variable feeder and drinker space. Birds were fed a commercial 5-phase diet in specific quantities per bird (Jhetam et al., 2022) and supplemental feeders and drinkers were provided throughout the first 10 d. Diet changes were made when the ration was finished, and feed amounts were adjusted for mortality.

Room temperature was set at 28°C for the first wk with the addition of heat lamps over the brooder rings for the first 10 d. Temperature decreased by approximately 1°C each wk to a target temperature of 16°C by wk 11. LED (light emitting diode) bulbs were used as the light source with daylength and light intensity starting at 23L:1D and 40 lux, and gradually reducing to a final daylength of 18L:6D at 5 lux by d 9. A 15-min dawn and dusk period was implemented throughout the trial by gradually increasing or decreasing light intensity prior to lights turning on or off. Intact straw bales were provided as environmental enrichment devices (1 bale/90 birds). As bales deteriorated, the straw was spread throughout the room and the bale was replaced.

One of the major aims in this study was to match air quality between treatments and minimize the effects of air quality on the hens to show the main effect of SD on birds’ health and wellbeing without the confounding factor of air quality. Air quality was closely monitored and controlled from d 1 and humidifiers were added during the first 7 d to maintain relative humidity (**RH**) at approximately 50% (Aviagen, 2015b). Carbon dioxide (**CO_2_**) was measured 3 times per wk using a handheld CO_2_ meter (CO_2_40; Extech Instruments; Nashua, NH) and ammonia was monitored once per wk until differences of 1ppm were detected, then measured twice per wk using ammonia Dräger-Tubes and a handheld pump (Draeger, Inc.; Houston, TX). If CO_2_ levels varied by 20% or ammonia differed by 5 ppm between rooms, ventilation was increased or decreased accordingly in each room independently to match air quality and temperature across all rooms (Beaulac and Schwean-Lardner, 2018; Beaulac et al., 2019). The ammonia and CO_2_ results from this study were previously reported (Jhetam et al., 2022). Room temperatures and RH were monitored hourly using iButton Hygrochron temperature and humidity data loggers (Maxim Integrated; San Jose, CA). Average weekly room temperatures (Jhetam et al., 2022) and RH did not differ between treatments (Table 2).

**Table 2:** Average weekly temperature (from Jhetam et al., 2022) and relative humidity across estimated final stocking density treatments from 1 to 11 weeks.

Age (weeks)	n	Stocking density (kg/m^2^)				SEM^1^	*P*-value^2^ (ANOVA)
		30	40	50	60		
**Temperature (°C)**							
1	4	28.2	28.3	28.3	28.3	0.08	0.98
2	4	27.4	27.4	27.2	27.3	0.08	0.90
3	4	26.0	26.0	25.7	25.8	0.09	0.69
4	4	24.1	23.9	23.9	23.9	0.06	0.65
5	4	22.4	22.3	22.1	22.2	0.07	0.63
6	4	20.9	20.8	20.7	20.7	0.11	0.96
7	4	19.9	19.5	19.6	19.6	0.09	0.44
8	4	19.1	19.1	18.8	19.0	0.06	0.36
9	4	18.6	18.4	18.4	18.5	0.07	0.88
10	4	17.7	17.6	17.3	17.3	0.12	0.63
11	4	16.4	16.5	16.4	16.5	0.08	0.96
**Relative Humidity (%)**							
1	4	54.9	53.7	54.6	53.9	0.09	0.87
2	4	52.8	53.8	53.4	51.0	1.09	0.46
3	4	55.9	58.3	57.3	55.7	0.92	0.62
4	4	47.1	46.4	47.5	45.9	0.18	0.55
5	4	44.4	45.4	43.6	44.0	1.09	0.39
6	4	56.4	59.4	57.6	60.3	0.86	0.09
7	4	62.3	59.9	64.8	63.4	0.93	0.48
8	4	58.2	60.6	57.5	61.2	0.21	0.08
9	4	58.7	52.0	57.1	59.9	1.09	0.10
10	4	62.1	64.8	59.5	63.6	1.12	0.22
11	4	61.5	63.7	61.9	64.8	0.23	0.19

1 Standard error of the mean.

2 ANOVA considered significant if *P* ≤ 0.05.

Mortality and morbidity were monitored twice daily (morning and afternoon), and birds were sent for necropsy to an independent pathology lab for identification of cause of illness or death. Birds were culled when necessary due to illness and/or skeletal or growth abnormalities. During the first 3-d, all mortalities and culls were replaced in an attempt to maintain the final predicted SD.

In trial 1, additional space was blocked off at wk 3 to account for high mortality rates during the first 3 wk of the trial, however, at wk 8 space was re-opened to maintain the estimated final SD for each treatment. Due to low mortality rates in trial 2, birds were removed from each treatment at wk 9 to prevent exceeding the final predicted SD at wk 11.

### Data Collection

Bird mobility was assessed using a gait scoring technique modified from broilers (Garner et al., 2002) for turkeys by Vermette et al. (2016) (Table 3). A total of 30 birds per replicate were randomly selected and gait scored at 8 and 11 wk of age by 2 trained observers. The 2 scores were then averaged for each bird. Footpad lesion scores were assessed from the same subsample of 30 birds per replicate at wk 8 and wk 11. Footpad lesion scoring was conducted by a trained individual using a method developed by Hocking et al. (2008), as shown in Table 4.

**Table 3:** Gait scoring technique (modified from broilers (Garner et al., 2002) for turkeys by Vermette et al. (2016.),

Score	Degree of impairment		Description
0	None	Original	Smooth, fluid locomotion. The foot is furled while raised.
		Modified	Straight legs.
1	Detectable, but unidentifiable abnormality	Original	Bird is unsteady or wobbles when walking, problem leg is unclear, or cannot be identified in the first 20 s of observation. Bird readily runs from the observer in the pen. Foot may remain flat when raised, the rest of the stride is fluid and appears unimpaired.
		Modified	Gait appears unstable (shaky or stomping)
2	Identifiable abnormality, that has little impact on overall function	Original	Leg producing the gait defect can be identified within 20 s of observation. If a problem leg is identified after 20 s of observed locomotor behavior, then the bird is classed as gait score 1. However, the defect has only a minor impact on biological function. Thus, the bird will run from the observer spontaneously or if touched or nudged. If the bird does not run at full speed, it runs, walks or remains standing for at least 15 s after the observer in the pen has ceased to move toward or nudge it. Birds in this, and previous, scores are often observed to scratch their face with their feet-again indicating little impact on function. (The most common abnormality in this score is for the bird to make short, quick, unsteady steps with one leg, where the foot remains flat during the step.)
3	Identifiable abnormality which impairs function	Original	Bird will move away from the observer when approached or touched, or nudged, it will not run, and squats within 15 s or less of the observer in the pen ceasing to approach or nudge it. If the bird squats after 15 s have elapsed, it is classified as gait score 2.
4	Severe impairment of function, but still capable of walking	Original	Bird remains squatting when approached or nudged. This is assessed by approaching the bird, and if it remains squatting, gently nudging or touching the bird for 5s. Bird may appear to rise but still rests on their hocks. Only rising to stand on both feet within 5s of handling is counted—a bird which takes longer than 5s to rise, or which does not rise at all is scored as 4, while a bird that rises in 5s or less is counted as a 3 (or lower if its gait is good). Nevertheless, the bird can walk when picked up by the observer and placed in a standing position, but squats immediately following 1-2 steps. (Squatting often involves a characteristic ungainly backwards fall.)
		Modified	Bird requires wings for balance.
5	Complete lameness	Original	Bird cannot walk, and instead may shuffle along on its hocks. May attempt to stand when approached but is unable to do so, and when placed on feet unable to complete a step with one or both legs.

**Table 4:** Footpad dermatitis (FPD) scoring system (Hocking et al., 2008).

Score	Description of Footpad
0	No external signs of FPD. The skin of the footpad feels soft to the touch and no swelling or necrosis is evident.
1	The pad feels harder and denser than a non-affected foot. The central part of the pad is raised, reticulate scales are separated, and small black necrotic areas may be present.
2	Marked swelling of the footpad. Reticulate scales are black, forming scale shaped necrotic areas. The scales around the outside of the black areas may have turned white. The area of necrosis is less than one quarter of the total area of the footpad.
3	Swelling is evident, and the total footpad size is enlarged. Reticulate scales are pronounced, increased in number and separated from each other. The amount of necrosis extends to one half of the footpad.
4	As score 3, but with more than half the footpad covered by necrotic cells.

Using the same subsample of birds, feather cover and cleanliness were assessed. Feather cover was scored on 5 areas of the body (neck, breast, wings, tail, and back) by 1 scorer on a scale from 1 to 4 adapted from Davami et al. (1987) and Sarica et al. (2008). A score of 1 indicated no feather cover, 2 indicated more than 50% of the plumage was missing, 3 indicated <50% of the plumage was missing, and 4 indicated that the bird had full intact plumage. Total feather cover was expressed as the sum of the 5 areas of the body evaluated for a maximum score of 20. Feather cleanliness was scored by 1 observer on a scale from 1 to 4 adapted by Forkman and Keeling (2009) from the broiler scoring system developed by Wilkins et al. (2003). A score of 1 indicated very clean (>75% of body feathers were free from soiling), 2 indicated moderately clean (50%–75% of body feathers were free from soiling), 3 indicated moderately dirty (25%–50% of body feathers were free from soiling), and 4 indicated very dirty (<25% of body feathers were free from soiling).

Birds that were targets of feather pecking and aggression that sustained mild open wounds were treated on the affected area with pine tar, a deterrent to birds that has antimicrobial properties (Barnes and Greive, 2017). All pine tar treatments were recorded, including the area of the body that was treated, to determine incidence of aggressive damage by location on the body and by time period. Birds that had more severe wounds were recorded as cull birds and placed in a hospital pen.

H/L ratio data were used to measure chronic stress in the birds. Blood was collected from a subsample of 20 birds per replication at 3, 5, 8, and 11 wk of age from the brachial vein into tubes containing EDTA anticoagulant using vacutainers. Blood smears were prepared on the same day blood was collected. After drying for 24 h, slides were stored in slide boxes, then stained after each trial. Slides were stained with PROTOCOL Hema 3 (Fisher Scientific; Ottawa, Canada) and stored in slide boxes until they were read. H/L ratios were determined by counting the number of heterophils and lymphocytes within a field of view (Thaxton et al., 2006; Beaulac and Schwean-Lardner, 2018) under 100X oil magnification until a total number of 100 cells were reached (microscope B-290TB; Optika©; Bergamo, Italy).

Bird activity was recorded using infrared video cameras (Panasonic WV-CF224FX; Panasonic Corporation of North America, Secaucus, NJ) located on the ceiling in each room. Video recordings were taken over a 24-h period at wk 8 and 11 in each room for both trials. Field of view observations were performed by an individual observer (video playback via Genetec Omnicast Software, Genetec Inc., Montreal, Canada) using an instantaneous scan sampling technique at 20-min intervals and the number of birds within the field of view performing each behavior was recorded (Torrey et al., 2013; Beaulac and Schwean-Lardner, 2018). Behaviors observed included those falling into the categories of mobility, comfort and maintenance, exploratory, nutritive, disturbances, and aggression, as defined in the ethogram (Table 5).

**Table 5:** Behavioural ethogram for turkey hens, as modified from Martrenchar et al. (1999) and Vermette et al. (2016),

Behavior	Description of Behavior
Feeding	Standing or sitting with head in the feeder.
Drinking	Standing or sitting with head in the drinker.
Resting	Lying down, not performing any other behaviour. May or may not be sleeping.
Standing	Standing, not performing any other behaviour.
Walking	Bird walking or running. Must take 2 or more consecutive steps.
Fighting	Two or more individuals, where at least one bird is posturing with head back and breast thrust forward. May or may not include one individual running or jumping at the other.
Preening	Manipulating own feathers with the beak while standing or resting.
Stretching	Extension of the wings and/or legs.
Wing Flapping	Flapping both wings.
Dust Bathing	Fluttering movement of the bird in a lying position on the litter while pulling the loose substrate close to the body and into the feathers.
Feather Ruffle	Full body shake while standing or resting.
Environmental Pecking	Pecking at walls, feeder tubes (not feed pan), drinker lines (away from the drinker cups), or litter while standing or resting.
Litter Pecking	Pecking at the litter while standing or resting
Feather Pecking	Pecking at a pen mate's feathers while standing or resting. The pen mate typically does not move away.
Aggressive Pecking	Forceful pecking at a pen mate's head, body, or snood while standing or resting. The pen mate typically moves away.
Moderate Disturbance	A bird in a laying posture opens its eyes, lifted its head or moved its body as a result of another bird walking in front of it, on top of it, touching it, or flapping near it.
Severe Disturbance	A bird in a lying posture stands up as a result of another bird walking in front of it, on top of it, or flapping near it.

Litter samples were collected at wk 8 and 11. Three samples per room were collected from a 10 × 10 cm area from the top of the litter to the floor below for each time point. Samples were taken from the front, middle, and back of the room. Samples were not taken from directly below the feeder or drinker lines. Litter was placed into paper bags and weighed before freezing at −18°C until all samples were ready for drying. The samples were placed in an oven (1330GSM Safety Oven; VWR Scientific; Plainfield, NJ) at 134°C for 36 h, after which samples were reweighed to calculate moisture content.

### Statistical Analyses

The experimental design was a randomized complete block design (trial as block) with rooms as the replicate unit. In order to meet normality (checked using Univariate Procedure in SAS9.4) and homogeneity of variances assumptions, behavior, footpad scores, gait score, feather cover and cleanliness scores, and incidence of aggressive damage data were log + 1 transformed and means and regression equations were then back transformed. Data from both trials of this study were analyzed together using regression analyses in SAS (SAS9.4, Cary, NC) via the Regression Procedure (Proc Reg) and Surface Response Regression Procedure (Proc RSReg) to determine a relationship between SD and the health and welfare parameters being evaluated (Beaulac and Schwean-Lardner, 2018). An ANOVA was performed for relative humidity data using the Proc Mixed Procedure (SAS9.4, Cary, NC) with SD as the fixed variable and trial as a random variable. A Tukey's range test was used to separate means. If *P* ≤ 0.05, differences were considered significant and if *P* ≤ 0.10, trends were noted.

## RESULTS

### Mobility and Footpad Scores

At 8 and 11 wk of age, SD treatments did not impact turkey hen mobility (Table 6). At 8 wk of age, average footpad scores (Table 6) increased linearly with increasing density (1.43, 1.55, 1.86, 1.80 for SD treatments 30, 40, 50, and 60 kg/m^2^, respectively; *P* = 0.03), indicating more severe lesions at high SD. However, at 11 wk, SD did not impact footpad scores (*P*(linear) = 0.16).

**Table 6:** Effect of estimated final stocking density on average gait score,^1^ average footpad scores^2^, and average litter moisture at 8 and 11 wk of age.

Wk	n	Stocking density (kg/m^2^)				SEM^3^	*P*-value (linear)	*P*-value (quadratic)	Regression equation^4^
		30	40	50	60				
**Average Gait Scores**									
8	4	0.37	0.25	0.29	0.49	0.051	0.41	0.13	-
11	4	0.53	0.70	0.70	0.93	0.098	0.18	0.89	-
**Average Footpad Scores**									
8	4	1.43	1.55	1.86	1.80	0.075	0.03	0.52	Y = 0.014x + 1.03
11	4	2.02	2.18	2.60	2.83	0.223	0.16	0.93	-
**Litter Moisture**^5^**(%)**									
8	4	30.32	29.10	33.96	36.01	1.601	0.13	0.61	-
11	4	28.81	31.23	35.82	47.67	2.880	<0.01	0.08	Y = 0.61x + 8.35

1 Score of 0 = no impairment and 5 = complete lameness (adapted from Garner et al., 2002 by Vermette et al., 2016).

2 Score 0 is no external signs of a lesion and score 4 is greater than 50% of the footpad covered with necrotic cells (Hocking et al., 2008).

3 Standard error of the mean.

4 Regression considered significant if *P* ≤ 0.05.

5 Average moisture of samples taken from front, middle, and back of room.

### Feather Cover and Cleanliness

At 8 wk of age, total feather cover (sum of 5 areas; max score of 20) linearly decreased as SD increased (17.96, 18.06, 17.30, 16.78 for SD treatments 30, 40, 50, and 60 kg/m^2^, respectively; *P* < 0.01; Table 7). Overall, at 11 wk of age, total feather cover linearly decreased as SD increased (17.09, 16.01, 15.68, 14.63 for SD treatments 30, 40, 50, and 60 kg/m^2^, respectively; *P* < 0.01). The average feather cleanliness scores (higher value indicates poorer cleanliness) worsened in a linear manner as SD increased at 8 wk of age (1.69, 1.78, 2.64, 3.15 for SD treatments 30, 40, 50, and 60 kg/m^2^, respectively; *P* < 0.01). At 11 wk of age, the average feather cleanliness scores were 1.79, 2.37, 2.80, and 3.00 for SD treatments 30, 40, 50, and 60 kg/m^2^, respectively, which also shows that cleanliness worsened in a linear manner as SD increased at 11 wk of age (*P* < 0.01).

**Table 7:** Effect of estimated final stocking density on turkey hen overall feather cover (scale 1–4^1^) and cleanliness scores (scale 1–4^2^) at 8 and 11 wk of age.

Wk	n	Stocking density (kg/m^2^)				SEM^3^	*P*-value (linear)	*P*-value (quadratic)	Regression equation^4^
		30	40	50	60				
**Total feather cover score (sum of 5 areas)**^5^									
8	4	17.96	18.06	17.30	16.78	0.159	<0.01	0.13	Y = −0.043x + 19.46
11	4	17.09	16.01	15.68	14.63	0.263	<0.01	0.96	Y = −0.077x + 19.33
**Average feather cleanliness score**									
8	4	1.69	1.78	2.64	3.15	0.181	<0.01	0.33	Y = 0.052x − 0.038
11	4	1.79	2.37	2.80	3.00	0.129	<0.01	0.09	Y = 0.041x + 0.66

1 Score of 1=no feather cover, 2= greater than 50% of the plumage is missing, 3= less than 50% of the plumage is missing, and 4=full intact plumage (Davami et al., 1987; Sarica et al., 2008).

2 Score of 1 = very clean, 2 = moderately clean, 3 = moderately dirty, and 4 = very dirty (Forkman and Keeling, 2009 as modified from Wilkins et al., 2003).

3 Standard error of the mean.

4 Regression considered significant if *P*≤0.05.

5 Sum of 5 areas for a maximum score of 20: neck, back, wings, tail, breast; scored on a scale of 1–4.

### Incidence of Aggressive Damage

The overall percentage of birds treated with a deterrent by location and the percentage of birds treated by time period that included culls for aggressive damage can be seen in Table 8. The percentage of birds treated for aggressive damage on the tail (*P* < 0.01) and the head (*P* = 0.05) linearly decreased with increasing density. When evaluating by time period, during wk 5 to 8 and 8 to 11, incidence of aggressive damage including culls related to aggressive damage was highest in the lowest SD of 30 kg/m^2^ (linear; *P* < 0.01 for both). The total percentage of birds treated for aggressive damage from 0 to 11 wk of age linearly decreased as SD increased (7.88, 7.60, 4.05, and 2.67% for SD treatments 30, 40, 50, and 60 kg/m^2^, respectively; *P* < 0.01).

**Table 8:** Effect of estimated final stocking density on the incidence and location of aggressive damage and skin tears (% of birds placed) treated with a deterrent up to 11 wk of age.

Location	n	Stocking density (kg/m^2^)				SEM^1^	*P*-value (Linear)	*P*-value (Quadratic)	Regression Equation^2^
		30	40	50	60				
**% of birds treated with a deterrent by location**									
Tail	4	2.96	2.13	0.73	0.66	0.381	<0.01	0.51	Y = −0.083x + 5.37
Wing	4	0.76	0.90	0.41	0.74	0.158	0.52	0.86	
Back	4	0.08	0.06	0	0.04	0.027	0.48	0.58	
Neck	4	0.42	0.58	0.47	0.13	0.096	0.12	0.07	
Head	4	2.88	2.84	1.50	0.74	0.455	0.05	0.49	Y = −0.077x + 5.48
Snood	4	0.08	0.26	0.26	0.26	0.067	0.52	0.32	
Skin tear	4	0	0.26	0.16	0	0.056	0.84	0.07	
Total	4	7.20	7.02	3.53	2.58	0.829	<0.01	0.45	Y = −0.17x + 12.89
**% of birds treated with a deterrent plus all culls related to aggressive damage by time period**									
Wk 0–3	4	0	0	0	0.04	0.011	0.19	0.32	
Wk 3–5	4	1.95	2.19	1.19	1.09	0.240	0.09	0.82	
Wk 5–8	4	2.97	2.71	1.14	1.05	0.343	<0.01	0.77	Y = −0.073x + 5.26
Wk 8–11	4	3.47	2.71	1.71	0.48	0.506	<0.01	0.22	Y = -0.10x + 6.58
Wk 0–11	4	7.88	7.60	4.05	2.67	0.883	<0.01	0.34	Y = −0.19x + 14.18

1 Standard error of the mean.

2 Regression considered significant if *P* ≤ 0.05.

### Heterophil/Lymphocyte Ratio

Turkey hen H/L ratio was not affected by SD at 3 wk of age. At 5 wk of age, H/L ratios linearly increased as SD increased (0.76, 0.85, 0.88, 0.89 ± 0.017 for SD treatments 30, 40, 50, and 60 kg/m^2^, respectively; *P* < 0.01). At 8 wk of age, H/L ratio in hens demonstrated a quadratic relationship with the 40 and 30 kg/m^2^ treatments having the highest ratios (1.09, 1.40, 0.83, 0.89 ± 0.032 for SD treatments 30, 40, 50, and 60 kg/m^2^, respectively; *P* = 0.03). At 11 wk of age, H/L ratios were 0.87, 0.96, 1.13, 1.06 ± 0.021 for SD treatments 30, 40, 50, and 60 kg/m^2^, respectively (*P* < 0.01), demonstrating a quadratic relationship.

### Behavior

The behavior of turkey hens (percentage of birds performing various behaviors based on the number of birds within a field of view) at 8 wk of age is shown in Table 9. The percentage of birds present at the feeder linearly increased as SD increased (4.63, 3.81, 8.61, and 6.25%, for SD treatments 30, 40, 50, and 60 kg/m^2^, respectively; *P* = 0.04). The percentage of birds resting linearly increased as SD increased, with birds in the 60 kg/m^2^ treatment resting the most (*P* < 0.01). Therefore, the opposite effects were seen for standing and walking behavior, where the percentage of birds standing (*P* = 0.02), and walking (*P* < 0.01) linearly decreased with increasing density. The percentage of birds performing litter pecking linearly decreased with increasing SD (3.20, 1.21, 2.29, 0.97% for SD treatments 30, 40, 50, and 60 kg/m^2^, respectively; *P* = 0.03). The percentage of birds dustbathing was highest in the 50 kg/m^2^ treatment (quadratic; *P* < 0.01) and the percentage of hens’ head scratching also demonstrated a quadratic relationship with the 40 and 50 kg/m^2^ treatments having the most birds performing this behavior (*P* = 0.01), however, these are low incidence behaviors. The percentage of birds that experienced severe disturbances linearly increased as SD increased (*P* = 0.01). The total incidence of disturbances, which included moderate and severe disturbances, linearly increased with increasing density (*P* = 0.05). The percentage of hens fighting linearly decreased with increasing density (*P* = 0.01) and aggressive pecking behavior demonstrated a quadratic relationship with SD, with birds in the 30 kg/m^2^ treatment performing that behavior more (*P* = 0.02). The percentage of birds performing aggressive behaviors, which includes fighting and aggressive pecking, also demonstrated a quadratic relationship with SD, with the highest aggression observed in the lowest SD treatment (0.60, 0.34, 0.11, 0.19 % for SD treatments 30, 40, 50, and 60 kg/m^2^, respectively; *P* = 0.01).

**Table 9:** Effect of estimated final stocking density on percentage of turkey hens performing various behaviours (% of birds within the field of view) at 8 wk of age.

Behaviour	n	Stocking density (kg/m^2^)				SEM^1^	*P*-value (linear)	*P*-value (quadratic)	Regression equation^2^
		30	40	50	60				
Feeding	4	4.63	3.81	8.61	6.25	0.652	0.04	0.60	Y = 0.10x + 1.48
Drinking	4	2.85	2.69	2.43	1.69	0.259	0.18	0.80	-
Standing	4	15.01	14.48	13.71	12.30	0.451	0.02	0.90	Y = −0.09x + 17.88
Walking	4	10.06	6.30	8.25	5.11	0.504	<0.01	0.96	Y = −0.13x + 13.24
Resting	4	57.89	64.52	59.74	67.05	1.169	<0.01	0.54	Y = 0.23x + 52.08
Litter pecking	4	3.20	1.21	2.29	0.97	0.302	0.03	0.48	Y = −0.06x + 4.44
Environmental pecking	4	0.82	1.25	0.57	0.72	0.108	0.88	0.85	-
Feather pecking	4	0.51	0.51	0.43	0.49	0.031	0.91	0.98	-
Preening	4	2.82	3.37	2.47	3.21	0.167	0.32	0.53	-
Stretching	4	0.25	0.12	0.19	0.17	0.021	0.95	0.25	-
Wing flapping	4	0.29	0.23	0.29	0.17	0.031	0.51	0.67	-
Dustbathing	4	0.00	0.02	0.05	0.00	0.007	0.63	<0.01	Y = −0.16e^−3^x^2^ + 0.02x-0.30
Perching	4	0.06	0.00	0.00	0.12	0.027	0.37	0.11	-
Head scratching	4	0.01	0.07	0.06	0.03	0.009	0.49	0.01	Y = −0.21e^−3^x^2^ + 0.02x − 0.3 7
Feather ruffle	4	0.18	0.11	0.28	0.08	0.032	0.99	0.22	-
Fighting	4	0.30	0.16	0.00	0.07	0.038	0.01	0.12	Y = −0.86e^−2^x + 0.52
Aggressive pecking	4	0.30	0.18	0.11	0.13	0.028	0.05	0.02	Y = 0.34e^−3^x^2^ − 0.04x + 1.09
Moderate disturbances	4	0.39	0.26	0.27	0.27	0.033	0.81	0.84	-
Severe disturbances	4	0.50	0.63	0.47	1.17	0.101	0.01	0.06	Y = 0.02x − 0.14
*Total comfort & maintenace*^3^	4	0.73	0.55	0.87	0.45	0.060	0.81	0.25	-
*Total aggression*^4^	4	0.60	0.34	0.11	0.19	0.055	<0.01	0.01	Y = 0.86e^−3^x^2^-0.09x + 2.59
*Total disturbance*^5^	4	0.89	0.88	0.74	1.43	0.114	0.05	0.07	Y = 0.01x + 0.32

1 Standard error of the mean.

2 Regression considered significant if *P* ≤ 0.05.

3 **Total comfort and maintenance**: stretching, wing flapping, dustbathing, head scratching, and feather ruffling.

4 **Total aggression**: fighting and aggressive pecking.

5 **Total disturbance**: moderate disturbances and severe disturbances.

The behavior of turkey hens (percentage of birds performing various behaviors based on the number of birds within a field of view) at 11 wk of age is described in Table 10. The percentage of birds present at the feeder at 11 wk was not impacted by SD, however, the percentage of birds at the drinker demonstrated a quadratic relationship with SD (2.97, 3.98, 2.79, 2.34 % for SD treatments 30, 40, 50, and 60 kg/m^2^, respectively; *P* = 0.02). The percentage of birds resting followed the same trend as wk 8 where more birds were observed resting in the 60 kg/m^2^ treatment (linear; *P* < 0.01) and the percentage of birds walking was highest in the lowest SD of 30 kg/m^2^ (linear; *P* = 0.03). The percentage of hens feather pecking was 0.56, 1.22, 0.90, 1.04 % for SD treatments 30, 40, 50, and 60 kg/m^2^, respectively (linear; *P* = 0.01). The percentage of birds head scratching was highest in the 30 kg/m^2^ (linear; *P* = 0.02). The percentage of birds that experienced severe disturbances followed a similar trend to wk 8 in which severe disturbances linearly increased with increasing density (*P* = 0.02). At 11 wk of age, more birds were fighting at 30 kg/m^2^ (linear; *P* = 0.01) and the percentage of birds aggressive pecking and total aggression, demonstrated a quadratic relationship with SD (*P* = 0.02 and *P* = 0.01, respectively).

**Table 10:** Effect of estimated final stocking density on percentage of turkey hens performing various behaviours (% of birds within the field of view) at 11 wk of age.

Behaviour	n	Stocking density (kg/m^2^)				SEM^1^	*P*-value (linear)	*P*-value (quadratic)	Regression equation^2^
		30	40	50	60				
Feeding	4	5.37	7.70	5.30	4.89	0.637	0.64	0.34	-
Drinking	4	2.97	3.98	2.79	2.34	0.196	0.15	0.02	Y = −0.37e^−2^x^2^ + 0.30x-2.54
Standing	4	13.05	11.16	9.56	9.45	0.653	0.24	0.62	-
Walking	4	8.86	5.75	5.08	4.51	0.609	0.03	0.19	Y = −0.14x + 12.22
Resting	4	58.90	60.57	69.12	70.28	1.985	<0.01	0.67	Y = 0.43x + 45.50
Litter pecking	4	1.87	1.44	0.77	1.14	0.150	0.10	0.13	-
Environmental pecking	4	1.94	1.42	1.79	0.55	0.222	0.12	0.07	-
Feather pecking	4	0.56	1.22	0.90	1.04	0.087	0.04	0.09	Y = 0.01x + 0.42
Preening	4	4.75	4.28	3.26	3.69	0.209	0.17	0.23	-
Stretching	4	0.19	0.16	0.10	0.09	0.022	0.23	0.78	-
Wing flapping	4	0.21	0.22	0.34	0.15	0.034	0.73	0.10	-
Dustbathing	4	0.03	0.04	0.01	0.02	0.006	0.25	0.87	-
Perching	4	0.01	0.00	0.00	0.07	0.013	0.12	0.12	-
Head scratching	4	0.12	0.06	0.04	0.01	0.016	0.02	0.77	Y = −0.34e^−2^x + 0.21
Feather ruffle	4	0.12	0.15	0.08	0.17	0.017	0.17	0.28	-
Fighting	4	0.10	0.13	0.04	0.10	0.018	0.89	0.85	-
Aggressive pecking	4	0.32	1.12	0.23	0.68	0.118	0.48	0.74	-
Moderate disturbances	4	0.25	0.19	0.22	0.27	0.030	0.51	0.47	-
Severe disturbances	4	0.38	0.43	0.35	0.76	0.063	0.02	0.18	Y = 0.01x + 0.61e^−2^
*Total comfort & maintenance*^3^	4	0.66	0.62	0.58	0.43	0.049	0.51	0.41	-
*Total aggression*^4^	4	0.42	1.25	0.27	0.77	0.128	0.55	0.80	-
*Total disturbance*^5^	4	0.63	0.63	0.57	1.03	0.079	0.06	0.21	-

1 Standard error of the mean.

2 Regression considered significant if *P* ≤ 0.05.

3 **Total comfort and maintenance**: stretching, wing flapping, dustbathing, head scratching, and feather ruffling.

4 **Total aggression**: fighting and aggressive pecking.

5 **Total disturbance**: moderate disturbances and severe disturbances.

### Litter Moisture

At 8 wk of age, litter moisture was not affected by SD (Table 6). At 11 wk of age, litter moisture increased linearly as SD increased (28.81, 31.23, 35.82, 47.67% for SD treatments 30, 40, 50, and 60 kg/m^2^, respectively; *P* < 0.01).

## DISCUSSION

One of the objectives of this study was to minimize the impact of confounding factors such as air quality; thus, ventilation was adjusted to match air quality in all treatments which included maintaining CO_2_ and ammonia within acceptable levels. Litter moisture can be significantly affected by increased SD (Zuidhof et al., 1993) and ventilation rate which causes increases in CO_2_ and ammonia (Mayne, 2005). Litter moisture at wk 8 was unaffected by SD which was likely due to reducing the impact of air quality in this study. Average CO_2_ and ammonia levels in both trials were consistent between treatments (Jhetam et al., 2022). However, higher litter moisture at 11 wk in the 60 kg/m^2^ treatment may have been caused when maximum allowable levels of ammonia were reached because external ambient temperatures were extremely low, and ventilation had to be reduced (Jhetam et al., 2022).

It is documented that litter moisture is directly affected by ventilation and the ability for the air to dry the litter (Zuidhof et al., 1993, 1995; Martrenchar et al., 1997, 1999; Beaulac and Schwean-Lardner, 2018; Beaulac et al., 2019). High SD can also affect litter moisture, as more birds produce more fecal matter (Proudfoot et al., 1979; Noll et al., 1991). Litter moisture in this study, while not impacted at 8 wk, increased with increasing SD at wk 11. In comparison, Beaulac et al. (2019) found litter moisture to be highest in the moderate SD treatments of 40 and 50 kg/m^2^. The authors suggested that in addition to ventilation rate and excreta output, the ability for air to circulate and reach the litter to dry it can be a contributing factor to increased litter moisture. Toms in those moderate treatments rested more, therefore, there was not enough open space to dry the litter (Beaulac and Schwean-Lardner, 2018; Beaulac et al., 2019). This is supported in the current study as a larger percentage of birds were resting on wet litter at high SD which may have prevented the litter from drying due to restricted air flow. Thus, increased contact with wet litter at high SD may have contributed to the increased incidence of FPD.

Litter moisture can be one of the primary factors contributing to FPD. Footpad lesions are of concern for a number of reasons, including their potential to become an entry pathway for bacteria which may lead to synovitis (inflammation of the synovial membrane) and lameness (Clark et al., 2002). Weber Wyneken et al. (2015) found FPD to be painful to turkeys and the presence of FPD affected gait and behavior. However, the authors suggested that more research was required to specifically identify the extent of pain for different footpad scores. Martrenchar et al. (1999) and Beaulac and Schwean-Lardner (2018) found footpad lesions to worsen with increasing SD from 38.8 to 62.7 kg/m^2^ at 12 (hens) and 16 (toms) wk of age and from 30 to 60 kg/m^2^ at 10 and 16 wk of age (toms), respectively. In the current study, the effect of SD on footpad lesions at wk 8 and 11 are in accordance with the studies discussed above.

Previous studies have found gait scores to be poorer in 12-wk-old hens and toms and 16-wk-old toms reared at higher densities (60 kg/m^2^ and above) (Martrenchar et al., 1999; Beaulac and Schwean-Lardner, 2018). However, mobility was not affected by increasing SD in this study. While there were no differences in mobility at these ages, it is possible that mobility would be affected in older, heavier flocks. In broilers, high SD was found to reduce mobility when bird movement was restricted (Sørensen et al., 2000). At both 8 and 11 wk of age, behavioral observation showed that turkey hens were less active at high SD and rested more. Therefore, at higher densities, there is reduced floor space which hinders the bird's ability to be active and this could lead to reduced growth (Jhetam et al., 2022) and poor mobility at older ages.

Feather cover (Leeson and Morrison, 1978) and cleanliness are important for both thermoregulation and protection from scratches (Forkman and Keeling, 2009). Feather pecking negatively impacts a bird's wellbeing as the removal of feathers can be painful and it can lead to cannibalism if the skin tears (Gentle and Hunter, 1991). Feather cover worsened with increasing SD at both 8 and 11 wk. This same effect was observed in two previous studies, where feather cover was poorer at high SD (60 kg/m^2^) at 10, 12, and 16 wk in toms (Beaulac and Schwean-Lardner, 2018) and at 14 wk in hens and toms at 48 kg/m^2^ (Coleman and Leighton, 1969) suggesting that feather cover can be affected at younger ages, even before maximum SD is reached. Feather cleanliness has only been studied in relation to SD in one previous study (Beaulac and Schwean-Lardner, 2018). Feather cleanliness worsened with increasing SD over the course of the 16-wk trial with toms (Beaulac and Schwean-Lardner, 2018) and this same effect was observed in this study. When feathers come into contact with fecal matter and wet litter, the feathers become wet and dirty which may cause the birds to lose body heat more quickly and require more energy to maintain body temperature (Beaulac et al., 2018; Beaulac and Schwean-Lardner, 2018). In addition, at high SD a larger percentage of birds exhibited feather pecking behavior which may have occurred as a form of social preening (Savory, 1995). This form of gentle feather pecking can be exploratory and directed at food particles or debris on a pen mates feathers (Savory, 1995; Hughes and Grigor, 1996; Dalton et al., 2013). Thus, dirtier feathers observed at high SD may have contributed to the occurrence of this behavior.

Aggressive pecking causing damage to the skin, often referred to as injurious pecking, is a welfare concern and is common in commercial strains of turkeys (Martrenchar et al., 2001). Aggressive damage was highest at low SD in this study. A larger percentage of hens in the 30 kg/m^2^ treatment were also observed standing, walking, litter pecking, and environmental pecking and it is thought that the more active birds are at low SD, the more aggressive encounters they may participate in (Beaulac and Schwean-Lardner, 2018). Alternatively, more aggressive damage was observed in toms at high SD (Buchwalder and Huber-Eicher, 2004) and at low and high SD between 4 and 8 wk of age (Beaulac and Schwean-Lardner, 2018). Incidence of aggressive pecking and damage was found to be lower in hens compared to toms, who reach sexual maturity earlier (Denbow et al., 1984; Leighton et al., 1985; Buchwalder and Huber-Eicher, 2003; Dalton et al., 2013). Sex differences or age of the birds may explain why hens did not exhibit aggressive damage at higher SD and only at lower SD when birds were more active.

In this study, stress increased linearly with increasing SD at 5 wk of age. Beaulac and Schwean-Lardner (2018) also observed increased H/L ratios at high SD at 4 wk and suggested that SD or group size may impact turkeys’ stress response early in life and may have been associated with increased aggressive damage and aggression related mortality and culls at that age. At 8 wk of age, birds were more stressed in the 30 and 40 kg/m^2^ treatments which may be related to the higher incidence of aggression related mortality and culls (Jhetam et al., 2022) and aggressive damage observed from 5 to 8 and 8 to 11 wk of age at low SD. At wk 11, higher H/L ratios were observed in the 50 and 60 kg/m^2^ treatments, and this may have impacted final BW (Jhetam et al., 2022) as observed in broilers, due to the reallocation of resources from growth towards the increased immune response to stress (McFarlane and Curtis, 1989; McFarlane et al., 1989; Puvadolpirod and Thaxton, 2000). It should be noted that the H/L response to mild and moderate stressors results in increased heterophils, whereas severe stressors result in basophilia (increased number of basophils) and heteropenia (lower than normal levels of heterophils), thus, the H/L ratio may become unreliable depending on the degree of the stressor (Maxwell, 1993; Maxwell and Robertson, 1998). Therefore, evaluating additional health and welfare parameters in conjunction with H/L ratios could more accurately determine the effects of SD on turkey hen wellbeing.

Birds housed at high SD were further impacted by reduced floor space, as evidenced by more birds being disturbed by other pen mates. Similar results were observed for disturbances in turkey hens as early as 6 wk of age up to 12 wk of age at high SD (Martrenchar et al., 1999). Broilers housed at high SD have been observed to be less active and experience more disturbances when birds move to the feeders or drinkers and come into contact with or walk over one another (Martrenchar et al., 1997; Simitzis et al., 2012). With more birds resting, pen mates had to maneuver between each other to access resources, resulting in more disturbances. Thus, is it evident that the wellbeing of turkey hens housed at high SD may be affected as they were much less active and experienced more disturbances when resting in comparison to those housed at low SD.

Stocking density can impact the productivity and welfare of birds, both of which are important for the development of SD guidelines. It is evident that high SD can negatively impact the health and welfare of turkey hens raised to 11 wk of age under conditions with similar air quality. Birds housed at high SD (60 kg/m^2^) had an increased incidence of FPD and poorer feather cover and cleanliness which supports the hypotheses of this study when air quality was equalized between SD treatments. It was also hypothesized that high SD would result in decreased activity and mobility, increased aggression, and higher stress. The decreased activity and higher H/L ratios observed at high SD support this hypothesis; however, mobility was not affected by SD and aggressive behavior and damage was lowest at high SD. At low SD, there was a higher incidence of aggressive damage and aggressive behavior which was likely caused by increased activity. Therefore, low SD does not necessarily equate better welfare in all measured components despite improved production (Jhetam et al., 2022). In conclusion, high and low SD (30 and 60 kg/m^2^) had negative impacts on the health and wellbeing of turkey hens. Thus, moderate densities may be more ideal to achieve optimal bird health and welfare. However, balancing the impacts on production parameters and economic return is also important when forming SD recommendations. Additionally, these moderate SD recommendations are based on maintaining air quality (CO_2_ and ammonia) at or below allowable levels by managing ventilation and barn temperature.
